# Morphometry of pyramidalis muscle and its role in reconstructive surgeries: A cadaveric study

**DOI:** 10.12688/f1000research.132477.1

**Published:** 2023-05-11

**Authors:** Suhani Sumalatha, Sharanya Rao, Vrinda Hari Ankolekar

**Affiliations:** 1 Department of Anatomy, Kasturba Medical College Manipal, Manipal Academy of Higher Education, Manipal, Karnataka, 576104, India

**Keywords:** Abdominal muscles, Anterior abdominal wall, Pyramidalis, Rectus sheath

## Abstract

**Background:** The pyramidalis muscle is a tiny triangular-shaped muscle that is located in the anterior abdominal wall, which originates from the body of the pubis and pubic symphysis and is inserted into linea alba. This study aimed to measure the different parameters of the pyramidalis muscle in adult cadavers.

**Methods:** This study was carried out on 31 adults (26 males and five females) 10% formalin embalmed cadavers of both sexes from the Department of Anatomy at Kasturba Medical College, Manipal.

**Results:** The mean length of the right pyramidalis muscle was 64.44 ± 12.52 mm and the left pyramidalis muscle was 64.73 ± 12.81 mm. The mean width of the muscle was 15 ± 4.18 mm and 15.03 ± 3.52 mm on the right and left sides, respectively. The mean thickness of the muscle was 1.32 ± 0.55 mm and 1.4 ± 0.80 mm on the right and left sides, respectively. The distance between the umbilicus to pubic symphysis ranged from 130–192 mm and their mean was calculated to be 159.77 ± 15.36. The distance between the umbilicus and the apex of the muscle ranged from 72–123 mm.

**Conclusions:** The measured parameters like length, width and thickness may help the surgeons to locate the muscle during infra umbilical surgeries.

## Introduction

The pyramidalis muscle (PM) is a tiny triangular-shaped muscle that is located in the anterior abdominal wall in humans. ^1^ Muscle is present in the lower anterior part of the rectus abdominis muscle enclosed by the rectus sheath. The muscle originates from the anterior surface of the body of the pubis and pubic symphysis. Through the tendinous fibers, PM is fixed in the anterior part of the pubis and through the ligamentous fibers; it is attached to the pubic symphysis. Inferiorly PM has a broad base, as it ascends, the muscle becomes slender and gets inserted medially into linea alba. Insertion of muscle takes place halfway between the umbilicus and pubis.

It is believed that the action of the PM contracts the linea alba and strengthens the abdominal wall. ^2^^,^^3^ On the other hand, its absence does not tend to result in a functional loss. ^4^ As a result, authors believe it is a vestigial muscle left behind from the pouches of marsupials and monotremes. ^5^

In humans, the PM has been linked to the assumption of upright posture. ^6^ The PM flap can be used to treat tiny intractable lesions in the foot and ankle, such as chemical wounds and prolonged osteomyelitis. ^4^ The PM, when cryopreserved, can be used as a source of stem cells that can then be employed to treat post-prostatectomy stress urinary incontinence. ^7^ When performing a longitudinal incision for a standard caesarean section, the PM was used to locate the linea alba and midline. ^8^

The morphometric study on PM in the south Indian adult human population is less studied. So this study aimed to explore the various parameters of PM in adult cadavers.

## Methods

### Ethical approval

The protocol was approved by the Institutional Ethics Committee (IEC-617/2021) Kasturba Medical College and Kasturba Hospitals, Manipal on September 15, 2021. Written informed consent was given by the body donors for teaching and research when they were alive.

### Study design and data collection

**Figure 1.**
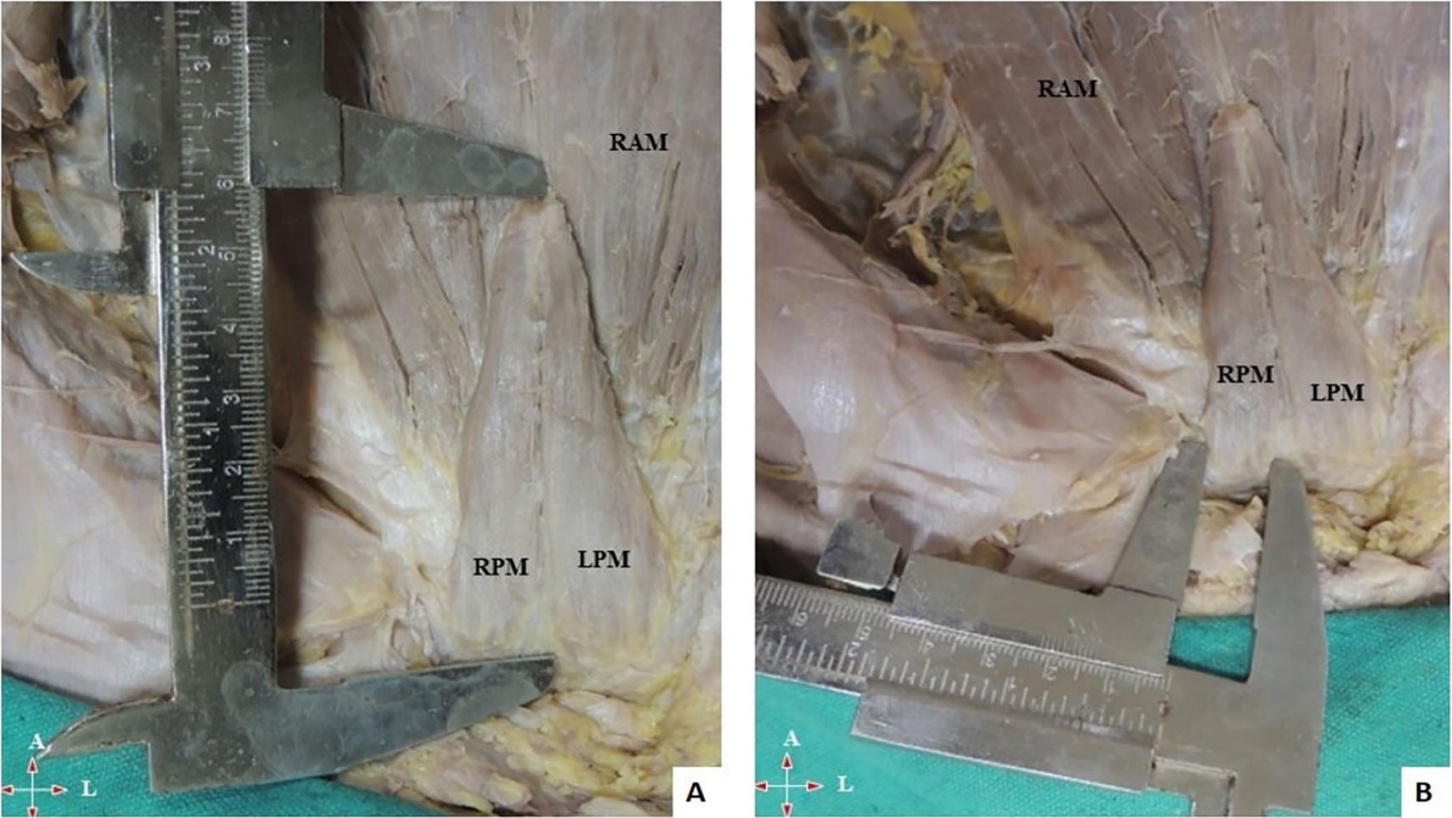
Anterior abdominal wall with PM. A) Measurement of length and B) width of PM near its origin. PM, pyramidalis muscle; RAM, rectus abdominis muscle; RPM, right pyramidalis muscle; LPM, left pyramidalis muscle; A, above; L, left.

**Figure 2.**
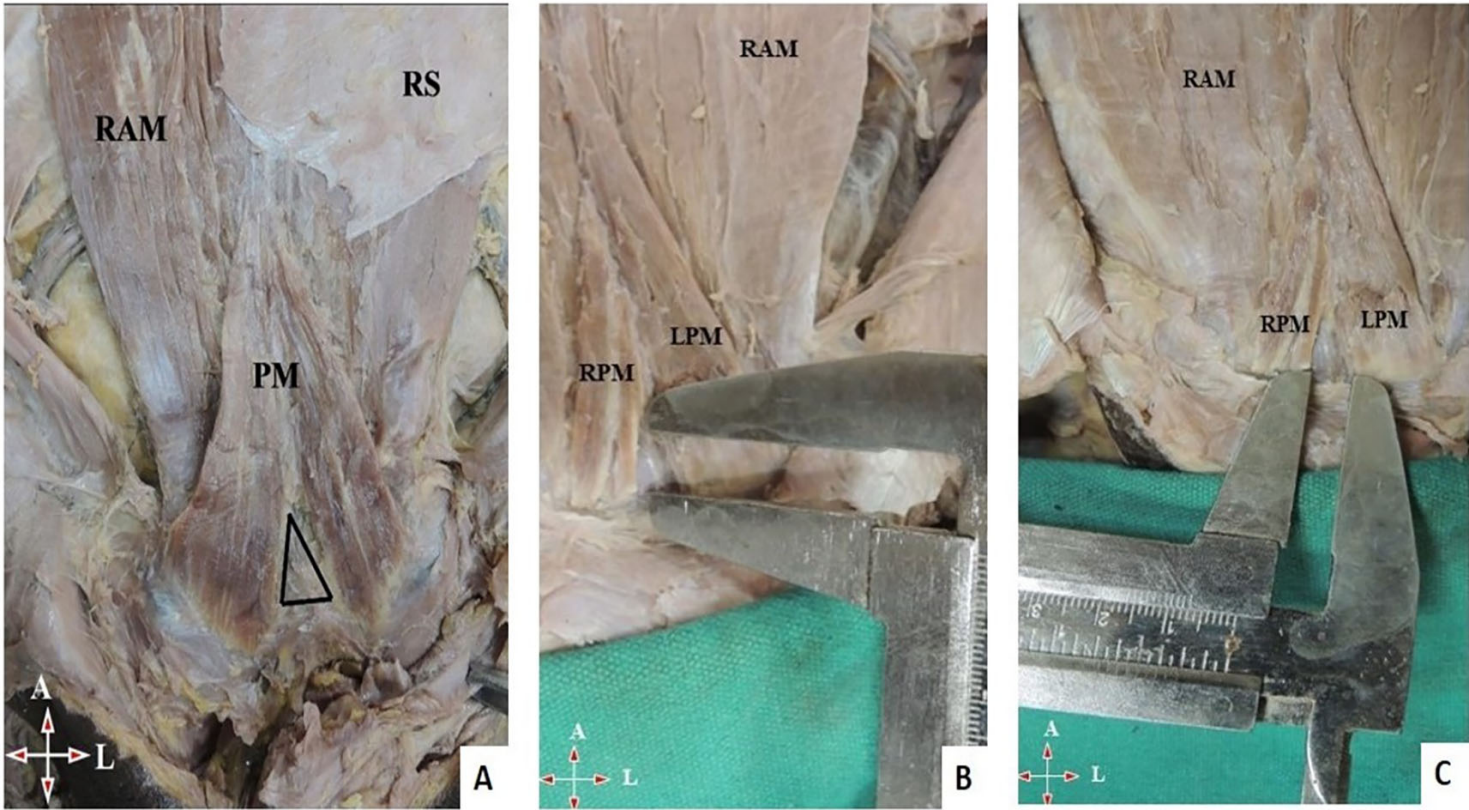
Anterior abdominal wall with PM. A) Gap between the PM. B) Measurement of length and C) width of gap between the PM. PM, pyramidalis muscle; RAM, rectus abdominis muscle; RS, rectus sheath; RPM, right pyramidalis muscle; LPM, left pyramidalis muscle; A, above; L, left.

**Figure 3.**
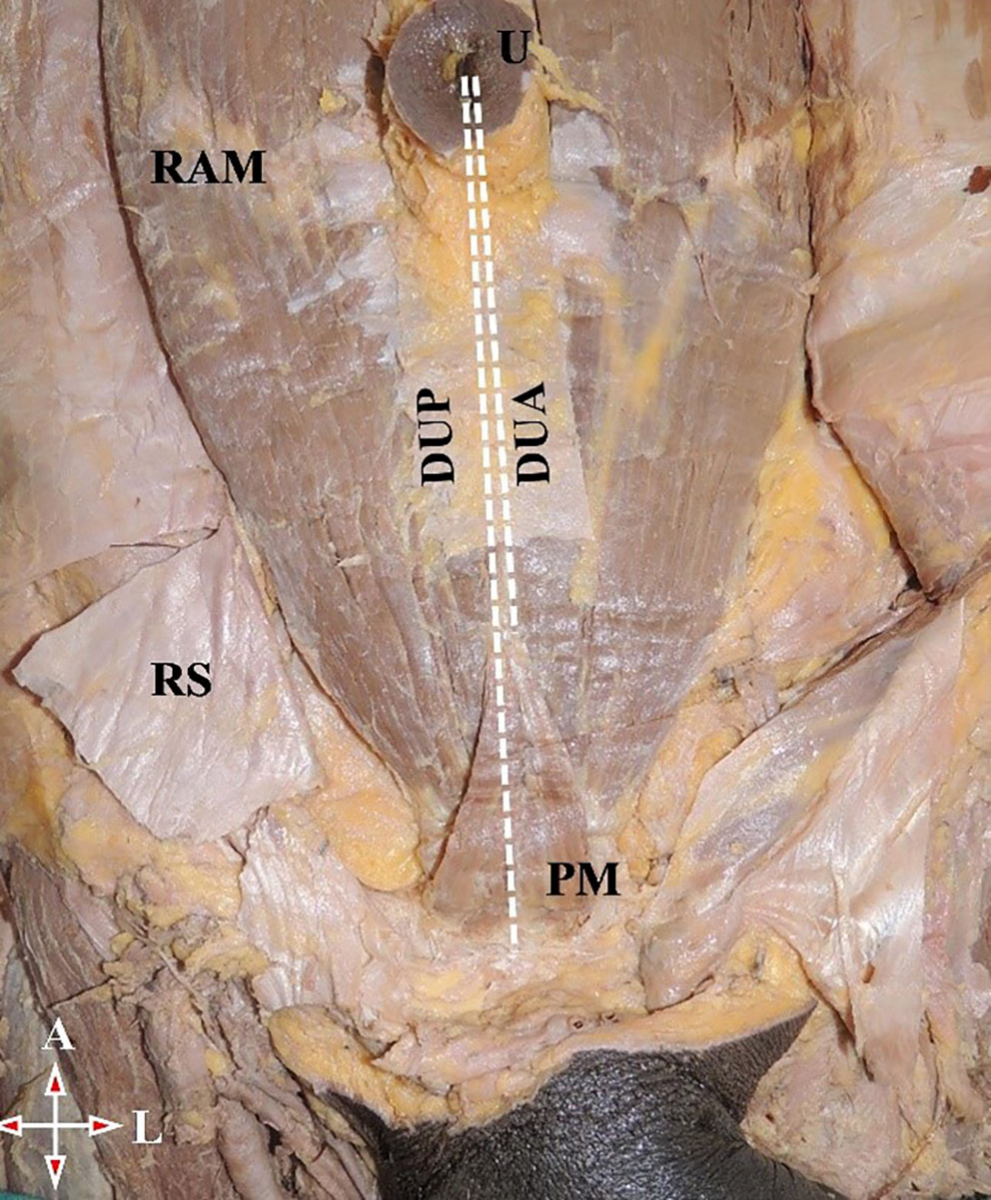
Anterior abdominal wall showing PM. PM, pyramidalis muscle; U, umbilicus; RAM, rectus abdominis muscle; DUP, distance between umbilicus to pubic symphysis; DUA, distance between umbilicus to apex of the muscle; RS, rectus sheath; A, above; L, left.

**Figure 4.**
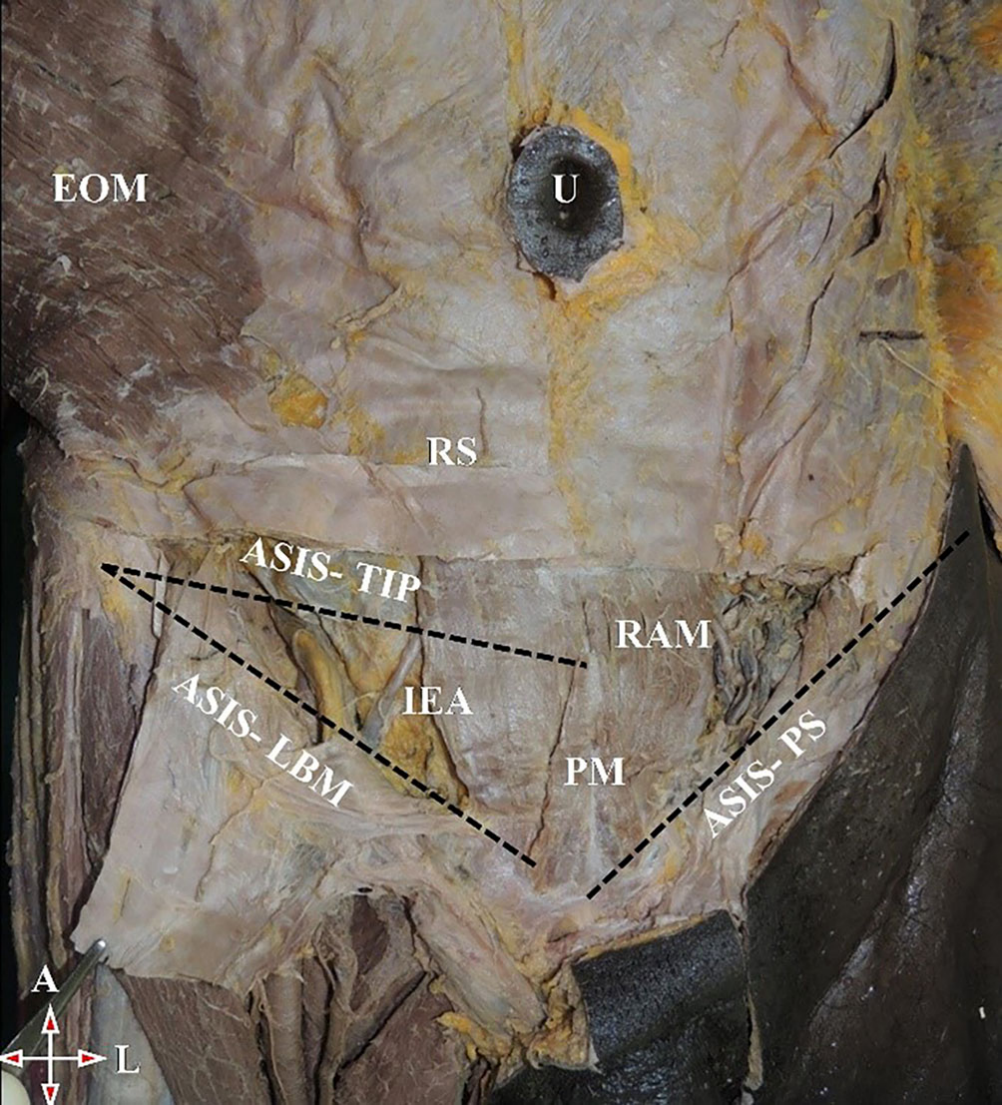
Anterior abdominal wall showing PM. ASIS-LBM: Distance between the anterior superior iliac spine to the lateral part of the base of the muscle. Distance between ASIS to tip of the muscle. Distance between ASIS and PS. PM, pyramidalis muscle; ASIS, anterior superior iliac spine; LBM, lateral part of the base of the muscle; PS, pubic symphysis; U, umbilicus; EOM, external oblique muscle; RS, rectus sheath; IEA, inferior epigastric artery; RAM, rectus abdominis muscle; A, above; L, left.

In this descriptive cross-sectional study, 31 adult cadavers of both sexes (26 males and five females) were utilized from the Department of Anatomy at Kasturba Medical College (KMC), Manipal for morphometric measurements. Data were collected between October 1, ^st^ 2021 to April 30 ^th^, 2022, for a period of six months. Available cadavers in the department were used for the study purpose with the sample size 31. It was a convenient sampling approach. Specimens with damage to the anterior abdominal wall were not considered for the study. With the surgical instruments, cadavers were dissected and muscles were exposed. The following parameters were measured using a vernier caliper:

• Length of muscle from the pubic symphysis to its attachment to linea alba (apex) along its medial border ( Figure 1A).
• Width at the base/origin of the muscle ( Figure 1B).
• The thickness of the muscle at the midpoint of the measured length.
• A small gap was found in few cadavers between right and left PM ( Figure 2A). The length and breadth of this gap were measured ( Figure 2B, Figure 2C).
• Distance between the umbilicus and pubic symphysis (DUP) and distance between the umbilicus and apex of the muscle (DUA) ( Figure 3).
• Distance between the anterior superior iliac spine (ASIS) and lateral part of the base of the muscle (LBM) ( Figure 4).
• Distance between ASIS to apex of the muscle ( Figure 4).
• Distance between ASIS and the pubic symphysis (ASIS-PS) ( Figure 4).

All the dimensions were completed by the same researcher to avoid inter observer variation.

### Data analysis

Descriptive statistics (mean, standard deviation, and range) were done for all measured parameters. To compare the right and left of each continuous variable, paired T-test was performed. P-value ≤ 0.05 was considered significant. The normally distributed data were correlated using Pearson's correlation coefficient analysis under the following combinations: i) Right and left PM length; ii) Right and left PM width; iii) Right PM length and right PM width; iv) Left PM length and left PM width; v) DUP and PM length (R and L); vi) DUP and PM width (R and L); vii) ASIS-PS (R) and PM width (R); viii) ASIS-PS (L) and PM width (L); ix) ASIS-PS (R) and PM length (R); and x) ASIS-PS (L) and PM length (L). SPSS (RRID:SCR_002865) version 16.0 (Chicago, SPSS Inc.) was utilized for data analysis.

## Results

### Morphometric measurements

*Descriptive statistics*

The length of the right PM ranged from 40–95 mm and the left PM ranged from 38–100 mm. The mean length of the right PM was 64.44 ± 12.52 mm and the left PM was 64.73 ± 12.81 mm. There was a 1–2 mm difference between the length of the right and left PM.

The width of the right PM and left PM ranged from 8–24 mm and 9–22 mm, respectively. The mean width of the right PM and left PM was 15 ± 4.18 mm and 15.03 ± 3.52 mm, respectively.

The PM thickness ranged from 0.5–2 mm on both sides. The mean thickness of muscle on the right side was 1.32 ± 0.55 mm and 1.4 ± 0.80 mm on the left side.

In 18 cadavers, a triangular gap was observed between right and left PM. Its length ranged from 8–26 mm and its width ranged from 5–17 mm.

Descriptive statistics of the remaining parameters are tabulated in Table 1. ^15^

**Table 1.** Overview of descriptive statistics calculated in adult cadavers (mm).

Variables	Mean ± Standard deviation	Range
Length (R)	64.44 ± 12.52	40–95
Length (L)	64.73 ± 12.81	38–100
Width (R)	15 ± 4.18	8–24
Width (L)	15.03 ± 3.52	9–22
Thickness (R)	1.32 ± 0.55	0.5–2
Thickness (L)	1.4 ± 0.80	0.5–4
Length of the triangular gap *	17.33 ± 5.30	8–26
Breadth of the triangular gap *	10.05 ± 3.60	5–17
ASIS - LBM (R)	122.5 ± 10.41	104–149
ASIS - LBM (L)	120.75 ± 8.00	105–138
ASIS - Apex (R)	118.25 ± 10.75	100–148
ASIS - Apex (L)	114.55 ± 12.16	94–144
ASIS - PS (R)	139.81 ± 7.74	122–158
ASIS - PS (L)	140.81 ± 7.10	128–152
DUA	97.48 ± 14.33	72–123
DUP	159.77 ± 15.36	130–192

R, right; L, left; ASIS, anterior superior iliac spine; LBM, lateral part of base of muscle; PS, pubic symphysis; DUA, distance between umbilicus to apex of muscle; DUP, distance between umbilicus to pubic symphysis.

* Length of the triangular gap as shown in Figure 2B; width of the triangular gap as shown in Figure 2C.

*Paired T-test*

A paired T-test was used to compare all the right and left dependent variables. ASIS-Apex (R) and ASIS-Apex (L) were significantly different from each other since the p-value was 0.0014. There was no significant difference between the two respective groups of other dependent variables.

*Pearson's correlation coefficient analysis*

Pearson's correlation coefficient analysis was done since the data were distributed normally. A significant positive correlation was observed between right and left PM length and right and left PM width. The above result suggests that the increase in length or width of the right PM was directly proportional to the length/width of left PM.

Correlation analysis between right PM length and width and left PM length and width showed a weak but positive correlation. From this, we can note that as the length of the muscle increases, the width of the muscle may or may not increase.

A weak correlation was observed when DUP and ASIS-PS was compared with the right and left PM length and width. From this, we can conclude that the DUP and ASIS-PS have negligible effects on the length and width of the PM. The correlation coefficient and its level, analyzed between the parameters are tabulated in Table 2.

**Table 2.** Correlation coefficient and level of correlation between the measured parameters in adults.

Correlated variables	Correlation coefficient	Level of correlation
Muscle length R & L	0.8824	Very strong positive
Muscle width R & L	0.7149	Strong positive
Right side muscle length and width	0.3411	Weak positive
Left side muscle length and width	0.2525	Weak positive
DUP & Muscle length (R)	0.3036	Weak positive
DUP & Muscle length (L)	0.2602	Weak positive
DUP & Muscle width (R)	0.2719	Weak positive
DUP & Muscle width (L)	0.1586	Very weak positive
ASIS-PS (R) & width (R)	0.0908	Very weak positive
ASIS-PS (L) & Width (L)	0.1841	Very weak positive
ASIS-PS (R) & length (R)	0.2315	Weak positive
ASIS-PS (L) & length (L)	0.2609	Weak positive

R, right; L, left; ASIS, anterior superior iliac spine; DUP, distance between umbilicus to pubic symphysis; PS, pubic symphysis.

## Discussion

Length and width of PM varies in different populations and it is tabulated in Table 3. It was observed in the present study, the length of PM in the female population was almost equal to the length of the PM reported by Natsis *et al.*, ^9^ Hojaij *et al.*, ^10^ and Kipkorir *et al.* ^11^ The width of the PM noted in the present study is almost equal to the study by Natsis *et al.* ^9^ A study by Das *et al.*, ^12^ reported the mean thickness of 32 ± 0.55 mm in the right PM and 1.4 ± 0.80 mm in the left PM. In our present study, the mean thickness of muscle on the right side was 1.32 ± 0.55 mm and 1.4 ± 0.80 mm on the left side, which was relatively less than the thickness reported in the study by Das *et al.* ^12^

**Table 3.** Morphometric measurements of pyramidalis muscle done in other studies.

Authors (ref.)	Year	Population	Pyramidalis measurements (mm)											
			Male						Female					
			Mean length			Mean width			Mean length			Mean width		
			R	L	R+L	R	L	R+L	R	L	R+L	R	L	R+L
Anson (8)	1938	American	-	-	68.2	-	-	19.8	-	-	-	-	-	-
Ashley-Montagu (1)	1939	White Americans	-	-	83.5	-	-	21.8	-	-	72.3	-	-	59.2
		African Americans	-	-	76.3	-	-	19.2	-	-	59.2	-	-	17.7
Kipkorir V (11)	2021	Kenyan	-	-	72	-	-	71.5	-	-	62.2	-	-	64.7
Kaur H (6)	2016	North Indian	49.7	49.7	-	17.5	17.2	-	48.7	47.8	-	12.5	14.5	-
Natsis K (9)	2016	Greek	83.7	75.0	-	16.1	15.6	-	61.8	65.6	-	15.0	15.5	-
Das SS (12)	2017	Indian	52.2	53.9	-	18.3	17.0	-	50.1	51.2	-	17.8	16.2	-
Hojaij FC (10)	2020	Brazilian	70.6	68.0	-	18.5	18.3	-	64.2	63.8	-	19.1	19.4	-
Present study	2023	South Indian (Male & female)	64.4	64.7	-	15.0	15.0	-	-	-	-	-	-	-

R, right; L, left.

In the present study, strong positive correlations were found between the lengths and widths of the PM on both sides, which is in line with the results of the study by Natsis *et al.* ^9^

The present study also measured ASIS-PS, ASIS-LBM, ASIS-Apex, DUP and DUA. The mean, standard deviation and range values of the aforementioned parameters are tabulated in Table 1. These parameters were not recorded in the previous studies; therefore we were not able to compare it with the other studies.

The present study also showed correlations between the DUP and PM length (R and L), DUP and PM width (R and L), ASIS-PS (R) and PM width (R), ASIS-PS (L) and PM width (L), ASIS-PS (R) and PM length (R), ASIS-PS (L) and PM length (L). The correlation analysis showed a weak but positive correlation between them. This suggests that increases or decreases in ASIS-PS and DUP have negligible effects on the length and width of PM.

Plastic surgeons and general surgeons must be aware of potential regional differences, to reduce the chance of consequences and implement the most effective therapeutic strategy. ^13^ A prostate-cystopexy employing PM for prostate stabilization can treat individuals with functional dysuria caused by the prostate's retrograde inclination and those with bladder obstruction caused by motor neuron injuries. ^14^

Therefore, it is essential for the surgeons to be aware of the morphometry of the pyramidalis muscle prior to making the different incisions needed for procedures involving the anterior abdominal wall.

### Implications

PM act as a landmark for surgeons during infra-umbilical and suprapubic incisions while performing caesarean sections. The PM may be collected through a reasonably unnoticeable Pfannenstiel (pubic) opening with minimal donor site complications making it a desirable donor muscle for microsurgical transfer.

### Limitations

The limitation of the present study is the smaller female cadaver sample size, with respect to the male cadaver sample size. Therefore a sex-based comparison was not performed in this study.

### Recommendations for further research

This study can be done to explore the foetus morphometric data and its clinical implications.

## Conclusions

The parameters like length, width, and thickness and new parameters such as ASIS-PS, ASIS-LBM, ASIS-Apex, DUP and DUA may help the surgeons to locate the muscle in the suprapubic region while performing surgical procedures to remove the flap of donor pyramidalis muscle.

## Data Availability

Figshare: Morphometry of pyramidalis muscle and its role in reconstructive surgeries: A cadaveric study. https://doi.org/10.6084/m9.figshare.22200181. ^15^ This project contains the following underlying data:
- EX sheet - Morphometric study of pyramidalis muscle.xlsx - STROBE-checklst.doc Data are available under the terms of the Creative Commons Zero “No rights reserved” data waiver (CC0 1.0 Public domain dedication).
