# Supplementary material for: Morphometry of pyramidalis muscle and its role in reconstructive surgeries: A cadaveric study in South Indian population
Source: F1000Res. 2024 Jan 8;12:484. Originally published 2023 May 11. [Version 3] doi: 10.12688/f1000research.132477.3 (PMC10558977; doi:10.12688/f1000research.132477.3)
Supplement: Peer review history

# Reviewer report: Reviewer response for version 3

**DOI:** 10.5256/f1000research.157860.r236181

**Published:** 2024-04-13

**Authors:** Mehtap Tiryakioglu Yuksel

**Affiliations:** 1 Near East University, Nicosia, TRNC Mersin, Turkey

The article is clear and ready to be indexed!

Is the work clearly and accurately presented and does it cite the current literature?

Yes

If applicable, is the statistical analysis and its interpretation appropriate?

I cannot comment. A qualified statistician is required.

Are all the source data underlying the results available to ensure full reproducibility?

Yes

Is the study design appropriate and is the work technically sound?

Yes

Are the conclusions drawn adequately supported by the results?

Yes

Are sufficient details of methods and analysis provided to allow replication by others?

Yes

Reviewer Expertise:

Anatomy

I confirm that I have read this submission and believe that I have an appropriate level of expertise to confirm that it is of an acceptable scientific standard.

# Reviewer report: Reviewer response for version 3

**DOI:** 10.5256/f1000research.157860.r236180

**Published:** 2024-01-31

**Authors:** Ranganath Vallabhajosyula

**Affiliations:** 1 Lee Kong Chian School of Medicine, Nanyang Technological University, Singapore, Singapore

The authors have addressed the queries raised by the reviewer.

Is the work clearly and accurately presented and does it cite the current literature?

Yes

If applicable, is the statistical analysis and its interpretation appropriate?

Partly

Are all the source data underlying the results available to ensure full reproducibility?

Yes

Is the study design appropriate and is the work technically sound?

Yes

Are the conclusions drawn adequately supported by the results?

Partly

Are sufficient details of methods and analysis provided to allow replication by others?

Yes

Reviewer Expertise:

Gross anatomy, histology, anatomical education, technologies in anatomy education,

I confirm that I have read this submission and believe that I have an appropriate level of expertise to confirm that it is of an acceptable scientific standard.

# Reviewer report: Reviewer response for version 2

**DOI:** 10.5256/f1000research.156562.r210635

**Published:** 2023-10-06

**Authors:** Ranganath Vallabhajosyula

**Affiliations:** 1 Lee Kong Chian School of Medicine, Nanyang Technological University, Singapore, Singapore

The authors have made a satisfactory attempt to revise and amend the manuscript. Indeed, the manuscript read well. 

 Comments: 

1. In the methodology, under study design and data collection, the authors have included hand-drawn images of measurements taken.  However, figure 1 does not display 1A and 1B, though the measurements taken were indicated with dotted lines and abbreviations.  It is suggested to indicate the hand-drawn image just as in Figure 1 showing the measurements - length (L) of the pyramidalis muscle (PM) from pubic symphysis (PS) to Linea alba (LA); and width (W) at the origin.  
2. The legend of Figure 1 should be modified as some of the features that are not indicated in the figure were listed.  for example, RAM is not shown in the image but indicated in the legend, and RPM and LPM are indicated in the legend, but not indicated in the image.  It is suggestible to concise the legend according to the content of the image shown.
3. Likewise for hand-drawn figure 2, instead of 2A, 2B, and 2C, it is advised to represent as one single figure 2, with proper annotations and revised legend.  Indicate only the structures shown in the image.  for example, in the legend, a - above; and b - below were indicated but not shown in the image, likewise RPM, RS, LPM, and RAM.
4. As indicated in comments 2 and 3, the legends for the remaining figures are to be modified.
5. If the authors were able to change the figure numbers, there numbers at the corresponding tables to be modified.

Is the work clearly and accurately presented and does it cite the current literature?

Yes

If applicable, is the statistical analysis and its interpretation appropriate?

Partly

Are all the source data underlying the results available to ensure full reproducibility?

Yes

Is the study design appropriate and is the work technically sound?

Yes

Are the conclusions drawn adequately supported by the results?

Partly

Are sufficient details of methods and analysis provided to allow replication by others?

Yes

Reviewer Expertise:

Gross anatomy, histology, anatomical education, technologies in anatomy education,

I confirm that I have read this submission and believe that I have an appropriate level of expertise to confirm that it is of an acceptable scientific standard, however I have significant reservations, as outlined above.

# Reviewer report: Reviewer response for version 2

**DOI:** 10.5256/f1000research.156562.r210633

**Published:** 2023-10-06

**Authors:** Shanu Markand

**Affiliations:** 1 A.T. Still University, Kirksville, Missouri, USA

The authors have addressed all comments from reviewers.

Is the work clearly and accurately presented and does it cite the current literature?

Yes

If applicable, is the statistical analysis and its interpretation appropriate?

Yes

Are all the source data underlying the results available to ensure full reproducibility?

Yes

Is the study design appropriate and is the work technically sound?

Yes

Are the conclusions drawn adequately supported by the results?

Yes

Are sufficient details of methods and analysis provided to allow replication by others?

Yes

Reviewer Expertise:

Cell culture, anatomical variaitions, histology, and vision sciences.

I confirm that I have read this submission and believe that I have an appropriate level of expertise to confirm that it is of an acceptable scientific standard.

# Reviewer report: Reviewer response for version 1

**DOI:** 10.5256/f1000research.145401.r184844

**Published:** 2023-08-17

**Authors:** Mehtap Tiryakioglu Yuksel

**Affiliations:** 1 Near East University, Nicosia, TRNC Mersin, Turkey

The article is providing a good morphometrical and statistical data of the pyramidalis muscle which can be an additional information for the readers who are in the field of medical sciences. 

 The cadaveric pictures are very clear to be read, yet a simply drawn diagram would also be a good addition to show the reference points for the measurements of the muscle-just a suggestion. 

 The title would be specified as "Morphometry of pyramidalis muscle and its role in reconstructive surgeries: A cadaveric study in South Indian population" 

 The embryology of pyramidalis muscle also could've been added as part of the Introduction as it may be absent in 20% of the people. Anatomical and morphometrical studies need a good emryological info, so the reason/s underlying the differences can be explained. Otherwise this is a good anatomical study to be indexed.

Is the work clearly and accurately presented and does it cite the current literature?

Yes

If applicable, is the statistical analysis and its interpretation appropriate?

I cannot comment. A qualified statistician is required.

Are all the source data underlying the results available to ensure full reproducibility?

Yes

Is the study design appropriate and is the work technically sound?

Yes

Are the conclusions drawn adequately supported by the results?

Yes

Are sufficient details of methods and analysis provided to allow replication by others?

Yes

Reviewer Expertise:

Anatomy

I confirm that I have read this submission and believe that I have an appropriate level of expertise to confirm that it is of an acceptable scientific standard, however I have significant reservations, as outlined above.

# Response

**Published:** 2023-09-25

**Authors:** Vrinda Ankolekar

**Affiliations:** Anatomy, Manipal Academy of Higher Education, Manipal, Karnataka, India

**Q1. The cadaveric pictures are very clear to be read, yet a simply drawn diagram would also be a good addition to show the reference points for the measurements of the muscle-just a suggestion.** 

 A. Four hand drawn diagrams to show the reference points for the measurements of pyramidalis muscle are added.

**Q2. The title would be specified as "Morphometry of pyramidalis muscle and its role in reconstructive surgeries: A cadaveric study in South Indian population"**

 A. The title is specified as above in the manuscript. 

**Q3. The embryology of pyramidalis muscle also could've been added as part of the Introduction as it may be absent in 20% of the people. Anatomical and morphometrical studies need a good emryological info, so the reason/s underlying the differences can be explained. Otherwise this is a good anatomical study to be indexed.**

 The embryology/ phylogeny of pyramidalis muscle is added as part of introduction.

# Reviewer report: Reviewer response for version 1

**DOI:** 10.5256/f1000research.145401.r184851

**Published:** 2023-08-17

**Authors:** Shanu Markand

**Affiliations:** 1 A.T. Still University, Kirksville, Missouri, USA

The current focused on the morphometric analysis of pyramidal muscle (PM).

**Strengths**

 The study design is good; the images are clear, and the results are interpreted thoroughly. Authors have been diligent in assessing all different aspects of PM morphometry. The paper is easy to follow. 

**Areas to Improve**

 The tile describes the pyramidalis muscles with its role in reconstructive surgeries. The paper covers the former part well, but the latter needs more discussion rather than a sentence or two. The authors need to comment on how different measurements can potentially relate surgically. 

 Was there any incidence of the unilateral presence of PM?

 Abbreviations should be avoided in the conclusions. 

 The triangular gap had a lot of variation, but this aspect was not discussed thoroughly. Additionally, what is the effect of embalming or quality of dissection and time passed after the dissection on these gap measurements (also other parameters)?

Is the work clearly and accurately presented and does it cite the current literature?

Yes

If applicable, is the statistical analysis and its interpretation appropriate?

Yes

Are all the source data underlying the results available to ensure full reproducibility?

Yes

Is the study design appropriate and is the work technically sound?

Yes

Are the conclusions drawn adequately supported by the results?

Yes

Are sufficient details of methods and analysis provided to allow replication by others?

Yes

Reviewer Expertise:

Cell culture, anatomical variaitions, histology, and vision sciences.

I confirm that I have read this submission and believe that I have an appropriate level of expertise to confirm that it is of an acceptable scientific standard, however I have significant reservations, as outlined above.

# Response

**Published:** 2023-09-25

**Authors:** Vrinda Ankolekar

**Affiliations:** Anatomy, Manipal Academy of Higher Education, Manipal, Karnataka, India

**Q1.The tile describes the pyramidalis muscles with its role in reconstructive surgeries. The paper covers the former part well, but the latter needs more discussion rather than a sentence or two. The authors need to comment on how different measurements can potentially relate surgically.**

 A. There is no literature regarding the importance of each and every parameters or how different measurements can potentially relate surgically. However these parameters can be useful for the plastic surgeons while giving abdominal incisions and removal of donor flap.

**Q2.Was there any incidence of the unilateral presence of PM?**

 Yes, in one cadaver

**Q3. Abbreviations should be avoided in the conclusions.**

 It is removed

**Q4. The triangular gap had a lot of variation, but this aspect was not discussed thoroughly. Additionally, what is the effect of embalming or quality of dissection and time passed after the dissection on these gap measurements (also other parameters)?**

 We used freshly embalmed cadavers and the dissections and measurements were done simultaneously. During suprapubic incisions, as well as while removing the donor flap,the knowledge of triangular gap between the pyramidalis muscle may be helpful for the plastic  surgeons.

# Reviewer report: Reviewer response for version 1

**DOI:** 10.5256/f1000research.145401.r173177

**Published:** 2023-07-14

**Authors:** Ranganath Vallabhajosyula

**Affiliations:** 1 Lee Kong Chian School of Medicine, Nanyang Technological University, Singapore, Singapore

There exists a good amount of literature on the morphometry of the pyramidalis muscle. The authors have made a decent attempt to add on the data to the existing information.

 The introduction, material and methods, results are well presented. 

 The results did not show whether the PM was observed in all the dissections and on both sides. In the results, the authors have reported a significant difference between the distance of ASIS to the TIP of PM on the right and left sides. It is suggestible to justify the reason for the significant difference. Did the authors observe any gender differences in the values, having less female sample though?

 The authors would have explained in more detail the importance of the Pearson correlation that was applied in their study and how these observations are useful for clinical interventions.

Is the work clearly and accurately presented and does it cite the current literature?

Yes

If applicable, is the statistical analysis and its interpretation appropriate?

Partly

Are all the source data underlying the results available to ensure full reproducibility?

Yes

Is the study design appropriate and is the work technically sound?

Yes

Are the conclusions drawn adequately supported by the results?

Partly

Are sufficient details of methods and analysis provided to allow replication by others?

Yes

Reviewer Expertise:

gross anatomy, histology, anatomical education, technologies in anatomy education,

I confirm that I have read this submission and believe that I have an appropriate level of expertise to confirm that it is of an acceptable scientific standard, however I have significant reservations, as outlined above.

# Response

**Published:** 2023-07-18

**Authors:** Vrinda Ankolekar

**Affiliations:** Anatomy, Manipal Academy of Higher Education, Manipal, Karnataka, India

**Q. The results did not show whether the PM was observed in all the dissections and on both sides**.

 A. PM was observed in 27 cadavers out of 31, in 26 cadavers it was bilateral and in one cadaver it was unilateral.

**Q.  In the results, the authors have reported a significant difference between the distance of ASIS to the TIP of PM on the right and left sides. It is suggestible to justify the reason for the significant difference**.

 A. The distance of ASIS to tip of right & left PM were significantly different because of the difference in the length of right and left PM.

**Q. Did the authors observe any gender differences in the values, having less female sample though?**

 A. We did not observe any gender differences

**Q. The authors would have explained in more detail the importance of the Pearson correlation that was applied in their study and how these observations are useful for clinical interventions**.

 A. The Pearson's correlation was performed to see whether there is any relationship between the measured values. We found strong correlation with length of right and left side PM. This could help the surgeons to locate PM and acquire required amount of flap to treat minor lesions in ankle region.
